# Perspective-Taking in Referential Communication: Does Stimulated Attention to Addressees’ Perspective Influence Speakers’ Reference Production?

**DOI:** 10.1007/s10936-018-9602-7

**Published:** 2018-09-15

**Authors:** Debby Damen, Per van der Wijst, Marije van Amelsvoort, Emiel Krahmer

**Affiliations:** Department of Communication and Cognition, Tilburg School of Humanities and Digital Sciences, Tilburg University, PO Box 90153, 5000 LE Tilburg, The Netherlands

**Keywords:** Perspective-taking, Referential communication, Egocentricity bias, Privileged information

## Abstract

In two experiments, we investigated whether speakers’ referential communication benefits from an explicit focus on addressees’ perspective. Dyads took part in a referential communication game and were allocated to one of three experimental settings. Each of these settings elicited a different perspective mind-set (baseline, self-focus, other-focus). In the two perspective settings, speakers were explicitly instructed to regard their addressees’ (other-focus) or their own (self-focus) perspective before construing their referential message. Results evidenced speakers’ egocentricity bias. Even though speakers were explicitly aware of addressees’ informational need, speakers still referred to information not known to their addressee. Speakers’ self-reported perspective-taking behavior correlated with their actual reference behavior. Those who reported to have regarded addressees’ perspective were also less likely to have leaked information about their own knowledge and attentional state. Findings are discussed in light of speakers’ egocentricity bias and the role of speaker-addressee collaboration in language production.

## Introduction

Engaging in successful referential communication implies that addressees are able to select the intended referent on the basis of speakers’ descriptions. For this, speakers are expected to design their message optimally (i.e., audience design in Clark and Murphy 1982) so that it adheres to addressees’ informational need (Clark 1992; Clark and Carlson 1982). Speakers are supposed to exchange just the right amount of information—neither too little nor too much—(Grice 1975) and base their contributions on the knowledge, beliefs and assumptions that are shared or salient between themselves and their addressee (i.e., common-ground information; Clark and Marshall 1981). This is necessary, because addressees will rely on this shared, salient knowledge when interpreting speakers’ reference (Arnold et al. 2013). Referential communication thus relies a great deal on interlocutors’ ability to accurately engage in the process of perspective-taking; the ability to take into account the knowledge and attentional state of their interaction partner at each step in the conversation. The questions that arise here are whether interlocutors are inclined to regard the other’s perspective accurately during interaction, and if this is not the case, whether a stimulated attention to another’s perspective would be beneficial for the referential communication process.

The literature shows a contradictory picture with regard to interlocutors’ ability and propensity to regard the other’s perspective during referential communication. On the one hand, it is argued that interlocutors are successful perspective-takers. In support of this view, studies have shown that addressees (e.g., Brown-Schmidt et al. 2008; Brown-Schmidt and Hanna 2011; Brown-Schmidt and Tanenhaus 2008; Hanna et al. 2003; Hanna and Tanenhaus 2004; Heller et al. 2008) as well as speakers (e.g., Heller et al. 2012; Nadig and Sedivy 2002) are able to rapidly integrate common-ground information (including their interlocutor’s perspective) from a very early stage in language comprehension and production processes. On the other hand, studies have shown that common-ground information (including the other’s perspective) is sometimes not fully integrated in the early stages of language processing, but only when interlocutors detect perspective-errors (e.g., Horton and Keysar 1996; Keysar et al. 1998, 2000, 2003). According to this view, interlocutors sometimes rely more on information that is not shared, but *privileged* to themselves, thereby disregarding the other person’s knowledge and attentional status (e.g., Keysar et al. 1998, 2000). These studies suggest that the production and comprehension of referential utterances are not necessarily constrained by the needs of the other person in the interaction, but more by one’s own knowledge and attentional status, resulting in perspective-judgments that are primarily based on information that is immediately accessible to oneself. In this sense, the other person’s knowledge is only considered in a later, optional stage in which interlocutors can choose whether to adjust their judgments to the common-ground status or not (Horton and Keysar 1996). These latter studies provide arguments for an interlocutor’s egocentricity bias (e.g., Keysar et al. 1998), demonstrating how interlocutors use their own mental state as a representational default to infer the one of their interaction partner (Epley et al. 2004). Engaging in perspective-taking is then considered to be a cognitive effortful process that can result in egocentric judgments when interlocutors do not correct their automatic response. Combining these two opposing views, research suggests that interlocutors integrate *both* common- and privileged-ground information during language processing, but that differences in interlocutors’ ability to inhibit their egocentric perspective might explain why egocentric biases sometimes prevail (e.g., Barr 2008; Brown-Schmidt 2009). Research has even shown that interlocutors are able to *switch* between visual self- and other perspectives, although perspective-switching requires cognitive effort and irrelevant perspectives might still interfere with accurate perspective-judgments (e.g., Apperly et al. 2010; Ferguson et al. 2017; Samson et al. 2010). Additionally, a more recent study sketches an even less pessimistic view on interlocutors’ egocentrism by suggesting that interlocutors might be flexibly egocentric (Samuel et al. 2018). Although an egocentric perspective may still have primacy, findings by Samuel et al. showed that once interlocutors adopted the other person’s perspective, they had a hard time to switch back to their egocentric default interpretation. Altogether, these findings suggest that interlocutors might be successful perspective-takers once they learn to inhibit an egocentric interpretation. Failure to suppress one’s own knowledge and attentional status during referential communication might lead to instances in which addressees sometimes select objects that are not visible to speakers (Apperly et al. 2010; Keysar et al. 2000, 2003; Legg et al. 2017), and speakers sometimes refer to information not known to their addressee (Horton and Keysar 1996) or even leak privileged information that should have stayed confidential (e.g., Kaland et al. 2011, 2014; Wardlow Lane et al. 2006).

In a referential communication task, Wardlow Lane et al. (2006) evidenced speakers’ informational leakage even when it had negative consequences. During the task, speakers described geometrical objects to their addressee, with the goal of earning both of them points if the addressee correctly identified the referent. Before every description, speakers hid one object from their addressees’ view. On critical trials, this object always differed in size from the target object speakers had to describe. On control trials, the hidden object differed in shape from the target object. Addressees could earn additional points by correctly guessing the identity of the hidden object. Although speakers were instructed not to let their addressee gain additional points, results showed that, on critical trials, speakers were very likely to cue the identity of their privileged object by referring to the size contrast they themselves were seeing. For instance, when speakers were instructed to hide a large square from addressees’ view and were subsequently asked to describe the target object that depicted a smaller square, speakers were very likely to indicate the size contrast they themselves were seeing by referring to the target as “*the small square*”. As addressees were only confronted with one square in common-ground, speakers’ inclusion of the size property of the target object was irrelevant and thus redundant. From addressees’ perspective, speakers’ reference thus contained too much information (Grice 1975), enabling addressees to use this redundant information to correctly guess the identity of speakers’ privileged object (Wardlow Lane and Liersch 2012).

Subsequent studies replicated findings of Wardlow Lane et al. (2006) by showing that speakers especially leak privileged information when this leakage is informative (Kaland et al. 2011, 2014; Wardlow Lane and Liersch 2012). Recall that on the critical trials in Wardlow Lane et al. (2006), speakers’ privileged object (a large triangle) and target object (a small triangle) were similarly shaped, but differed in size. On control trials, however, the privileged object (a small square) and the target (a small triangle) were presented in similar sizes, but in different shapes. This means that, only on the critical trials the target object’s size was meaningful to speakers. Therefore, on critical rather than on control trials, the contrast presented was salient to speakers, stimulating them to refer to the object’s size. Since referring to the size of objects is only relevant in relation to other objects, speakers did not refer to the size contrast on control trials. In a study that was inspired by Wardlow Lane et al. (2006), Kaland et al. (2011, 2014) presented speakers with size contrast on all trials. Kaland et al. (2011, 2014) showed that speakers were more likely to leak information about the target object’s size when the contrast between their privileged object and the target was meaningful (on salient trials: large triangle, small triangle) than when this was not the case (on non-salient trials: large square, small triangle). That is, speakers were more likely to refer to the “*small triangle*” when it was contrasted to a bigger triangle (salient trials) than to a bigger square (non-salient trials) in speakers’ privileged-ground. The informativeness of the size-contrast on critical rather than on control trials boosted its salience to speakers, making it more likely that speakers would refer to it. Kaland et al. further showed that this boost in salience did not affect object features that are inherently salient (such as color). That is, speakers were not more likely to refer to the object’s color when the color-contrast was informative (blue triangle, red triangle) than when it was not (blue square, red triangle).

Speakers are also very likely to leak information non-verbally, (Kaland et al. 2011, 2014), and especially when they do not have enough cognitive resources left to correct perspective mistakes (Wardlow Lane and Ferreira 2008). Intriguingly, speakers are even more likely to refer to privileged information when they are motivated to keep it confidential. The motivation to keep private information privileged further enhances its salience which, as a consequence, can ironically (Wegner 1994) result in a stronger tendency of it being revealed (Wardlow Lane and Liersch 2012). It seems that despite their efforts, speakers are not always able to monitor for perspective mistakes or to adjust their egocentric errors to addressees’ informational need. This raises the question whether speakers’ audience design would benefit from a constant reminder of addressees’ perspective.

Research investigating the influence of perspective-taking on reference comprehension has shown that addressees are less influenced by their privileged perspective if they are instructed to take the speaker’s perspective (Ferguson et al. 2017). With regard to reference production, research suggests that speakers are more likely to engage in an accurate audience design if they are (made) aware that such design is needed. For instance, evidence from eyetracking research shows that interlocutors engaging in perspective-taking are less influenced by privileged perspectives (Ferguson et al. 2017), and speakers who requested information from their addressee rather than informed them were more likely to adjust their references to the perspective of the addressee (Yoon et al. 2012), as were the speakers who had an interdependent focus—rather than an independent focus—deriving from their cultural background (Wu and Keysar 2007). Furthermore, research has shown that speakers learn through repeated experience how their references should be adapted to the informational need of the addressee (Horton and Gerrig 2002). In Horton and Gerrig (2002), speakers gained their experience by receiving feedback from their addressee about the informativeness of their reference. By means of this feedback, addressees cued the knowledge they required from their speaker. This is an interesting finding that also seems to suggest that speakers’ audience design benefits from an explicit attention to their addressees’ knowledge and attentional state. If speakers are able to adjust their reference production to their addressees’ perspective through repeated experience (Horton and Gerrig 2002), what will happen to speakers’ audience design if they explicitly attend to addressees’ informational need before they even start producing their reference? Guiding speakers through an explicit perspective-taking process before reference production might inhibit egocentric anchoring, and might stimulate speakers to monitor for perspective mistakes. This might incite speakers to correct for egocentric errors such as the leakage of privileged information (Horton and Keysar 1996), resulting in references that are more accurately based on addressees’ perspective, and less on speakers’ own knowledge and attentional state.

From a pragmatic point of view, it is interesting to investigate whether previously found egocentric errors (e.g., Horton and Keysar 1996; Kaland et al. 2011, 2014; Wardlow Lane et al. 2006, 2008, 2012), can be countered by an explicit mental activation of the others’ informational need. What if interlocutors in the abovementioned studies were made *explicitly* aware of the others’ perspective, would their reference production still be influenced by privileged information? This question is also interesting for social practices that try to enhance perspective-taking during social interaction, using explicit perspective-taking instructions (e.g., Brown 1997, 2010; Fleuridas et al. 1986; Penn 1982; Selvini-Palazzoli et al. 1980; Tomm 1985). This research provides a first step in investigating the fundamental role these explicit perspective-taking instructions can play during perspective-taking, and, in particular, the extent to which these explicit perspective-taking instructions stimulate speakers’ audience design during reference production.

## The Current Research

Two experimental studies examine the question whether speakers’ referential communication benefits from an explicit focus on addressees’ perspective. This question is investigated among student dyads taking part in a referential communication game in which they are randomly assigned the role of the speaker or addressee. In both experiments, we test the hypothesis that speakers are less likely to refer to information privileged to them when they are explicitly instructed to regard addressees’ perspective than when these explicit perspective-taking instructions are absent. The second experiment intensifies the perspective-manipulation used in experiment 1 and investigates the role of speakers’ self-versus other-awareness during perspective-taking. Results of both studies provide more insight in the role perspective-taking processes play during the production of referential descriptions, thereby providing further insights into when and how common-ground information is incorporated in the process of language production (e.g., Bezuidenhout 2013; Horton and Gerrig 2002).

## Experiment 1

Experiment 1 investigates whether speakers’ elicited attention to addressees’ perspective influences their reference production. Following the assumptions of the egocentricity hypothesis (Keysar et al. 1998), speakers in a natural communicative setting (i.e., baseline) are likely to anchor their referential expressions to their own knowledge and attentional state, increasing the likelihood they will refer to information that is privileged to them. We therefore expect that other-focused speakers, i.e., whose attention is explicitly focused on their interlocutor’s perspective, will be less likely to refer to privileged information than the speakers in a baseline setting. Furthermore, since speakers are expected to be naturally biased to anchor their reference production to their own perspective, we hypothesize that self-focused speakers, i.e., who are made explicitly aware of their own perspective, are even more likely to refer to privileged information than speakers referring in a baseline setting.

In addition to these expectations, we hypothesize that speakers’ egocentric anchoring will be influenced by the salience of speakers’ privileged information. As Wardlow Lane et al. (2006) and Kaland et al. (2011) have shown, the salience of speakers’ privileged knowledge can incite speakers to unintentionally refer to information they want to keep concealed. It is thus expected that, overall, speakers will refer more to privileged information when this information is salient versus non-salient to them. Since self-focused speakers are explicitly instructed to pay attention to the information that is available to themselves, we expect that these speakers will be influenced more by the salience of their private information than speakers without an induced perspective-focus (i.e., baseline). Compared to the speakers communicating in the baseline setting, we thus hypothesize that self-focused speakers will be more likely to leak privileged information when this information is salient than non-salient to them. This in contrast to the speakers with a stimulated other-focus. Since other-focused speakers are explicitly instructed to focus their attention on their addressees’ perspective, we expect that these speakers will be influenced less by the salience of their own knowledge and attentional state. That is, in contrast to speakers in the baseline setting, we hypothesize that other-focused speakers will be less likely to leak privileged information, regardless of its salience.

### Method

#### Participants

In total, 93 student-dyads (*n* = 186) participated in this study. The data of three dyads were excluded from analyses, due to an error in the experimental procedure (*n* = 2), or due to a low proficiency in the language of the experiment (Dutch) (*n* = 4). The analyses were thus based on 90 dyads in which the participants were randomly assigned either the role of the speaker (55 women, 35 men, *M*_*age*_ = 22.0 years; age range 18–34 years) or the role of the addressee (59 women, 31 men, *M*_*age*_ = 21.3 years; age range 17–27). All participants were fluent in Dutch, did not experience problems at discerning the colors used in the study, and received course credits for their participation.

#### Design

**Fig. 1:**
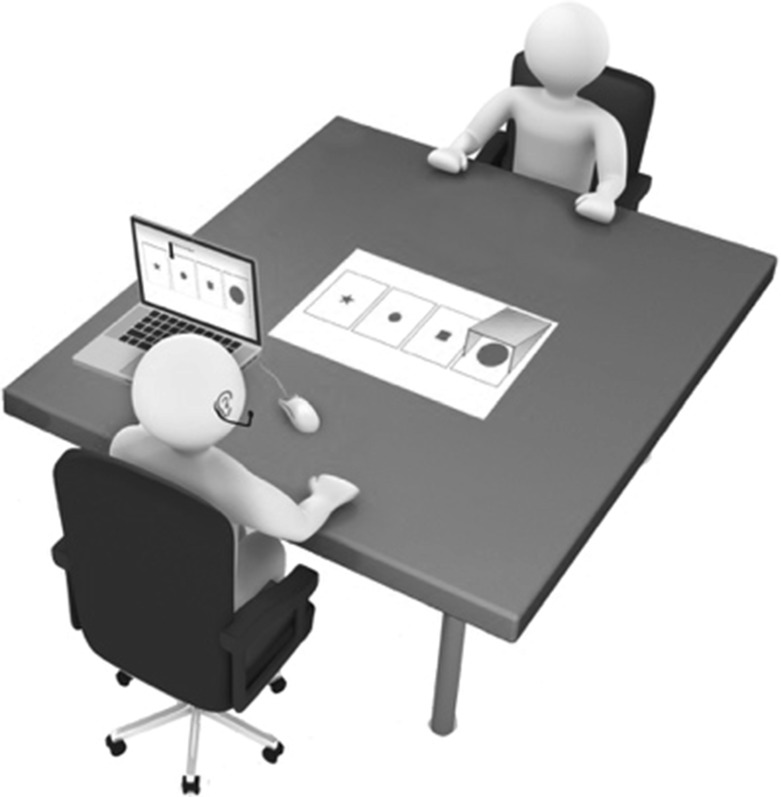
The experimental setting in which the speaker (on the bottom) identified figures to the addressee (on the top)

The experimental design and procedure were replicated from Kaland et al. (2011, 2014), which in turn were inspired by Wardlow Lane et al. (2006). The experiment consisted of a collaborative referential communication task in which speakers were asked to describe mutually visible geometrical figures in such a way that the addressee could indicate the intended one out of a set of four. Speakers and addressees were seated across from each other at a table. Out of these four figures, three were visible to both addressees and speakers, and one was privileged to speakers and thus hidden from addressees’ view. The three mutually visible figures were differently shaped (e.g., circle, triangle, square) and could thus be distinguished by mentioning their shape. A schematic of the four figures was physically presented on the table in front of both interlocutors. The same schematic was depicted on speakers’ private computer screen. From their private computer screen, speakers were instructed to block one figure from their addressee’s view and, subsequently, to identify another figure that was mutually visible to both speaker and addressee on the table in front of them (Fig. 1). The occluded figure differed either in size or color from the three mutually visible figures. In our experiment, we replicated Kaland et al. (2011, 2014) privileged situation and added a perspective-taking manipulation. In this privileged setting, one object was always blocked from addressee’s view and thus belonged to speaker’s privileged ground.

#### Materials

##### Eliciting Self- Versus Other-Focus

Speakers’ self- versus other-focus was manipulated by asking them explicitly to either regard their own (self-focus) or their addressees’ (other-focus) perspective before they identified the target object. Participants were randomly assigned to one of the three communication settings (self-focus, other-focus, baseline), resulting in 30 speakers per setting. The self- versus other-focus was operationalized by asking speakers to answer a perspective question portrayed on the computer screen next to them. In the self-focus setting, speakers answered the question reinforcing their egocentric perspective: “*Which four figures are visible to you?*”. This in contrast to the speakers in the other-focus setting who were asked to regard the perspective of their addressee: “*Which three figures are visible to your addressee?*”. Speakers answered the question by selecting the figures on their private computer screen. To eliminate the possibility that the self-focused speakers would simply select all figures as a response to the question, a fifth figure was added to the schematic presented on the computer screen. The fifth figure’s position and shape were balanced across all trials. To examine how speakers’ reference production in the self-versus other-focused setting diverged from a baseline situation, we allocated one-third of the speakers to a setting in which a self-versus other-focus was not reinforced.

##### Salience of Privileged Information

Participants were confronted with 40 trials, consisting of 20 salient and 20 non-salient trials. In the salient trials, speakers’ privileged figure was similarly shaped to the target figure to be identified, but differed from this target on one feature (size/color). In the non-salient trials, the privileged figure could be distinguished on two features (shape, size/color). For example, when the target figure depicted a small triangle, it could be contrasted to a privileged big triangle (salient trial) or to a privileged big circle (non-salient trial). The relation between the hidden and privileged figure is thus more salient when they differ on only one feature than on two. Successive figures were not similarly shaped, and half of the figures contrasted in size (big, small) and the other half in color (red, blue, green, black, grey, yellow). When the contrast was presented in size (small privileged triangle, large target triangle), all figures contained the same color. In turn, when the contrast was presented in color (red privileged triangle, blue target triangle), all figures contained the same size. We replicated the number of features (size/color) on which speakers’ privileged and the target figure showed a contrast, so that we were able to adhere as closely as possible to the original experimental design of Kaland et al. (2011, 2014). The figures’ shape, color, and position in the four-card schematic were balanced across all trials. This resulted in a 3 × 2 × 2 design, with the communicative setting (self-focus, other-focus, baseline) as a between subjects factor, and trial type (salient, non-salient), and contrast type (color, size) as within subjects factors.

#### Procedure

A role of the dice decided which participant took the role of the speaker. Participants were told that, when the addressee was able to correctly identify the target figure, both the speaker and the addressee would obtain one point. Participants were further told that failing to identify the target figure would result in zero points obtained, and that the goal of the game was to obtain the maximum number of points. Speakers and addressees were completely free during the interaction and did not receive additional instructions on how to play the game. That is, speakers were not told how to structure their reference and we left it to the addressees to decide whether or not they wanted to provide feedback.

Speakers and addressees sat down on opposite sides of a table. Speakers were seated next to a computer screen on which the experimental trials were presented using E-Prime version 2. At the beginning of each trial, addressees closed their eyes while the experimenter placed four cards on the table. Addressees’ eyes remained closed until the speaker identified the target. When the four cards were put in place, speakers (a) hid one figure from their addressees’ view by placing an occluder between the figure and their addressee. Subsequently in the other- and self-focused setting, speakers (b) answered a perspective question by selecting either the three figures visible to their addressee (other-focus) or the four figures visible to them (self-focus). Hereafter, speakers (c) described the target object. Speakers were instructed to look at the four cards on the table when referring to the target object. While hearing speakers refer to a figure, addressees opened their eyes and pointed at the intended figure on the table in front of them. Speakers subsequently (d) informed their addressee whether their selection was correct. Since speakers in the baseline setting were not confronted with a perspective-taking manipulation, these speakers only performed actions (a), (c), and (d).

The experimental game ended after 40 rounds. After the final round, speakers indicated on a 10-point scale to what extent they took into account their addressees’ perspective during the game (1 = *not at all*, 10 = *very much*). Addressees indicated on a 10-point scale how much the speaker had used redundant (i.e., size or color) information to describe the targets to them (1 = *not at all*, 10 = *very much*). Since audio recordings were made of all sessions,^1^ participants’ consent to making these recordings and using them for scientific purposes were collected. Afterwards, all participants were debriefed.

#### Coding

To measure speakers’ reference to privileged information (RPI), we counted the adjectives that matched the contrast (in size or color) between the target and privileged figure. Adjectives that did not contrast the target figure to the privileged one were not taken into account. For example, if speakers were to refer to the target as “the *small* triangle”, information about the object’s size was only considered as informational leakage when speakers’ privileged figure depicted a similarly shaped figure (i.e., a large triangle). Speakers’ RPI was calculated as a proportion (1 = *contrasting adjective uttered*; 0 = *no contrasting adjective uttered)*.

### Results

**Fig. 2:**
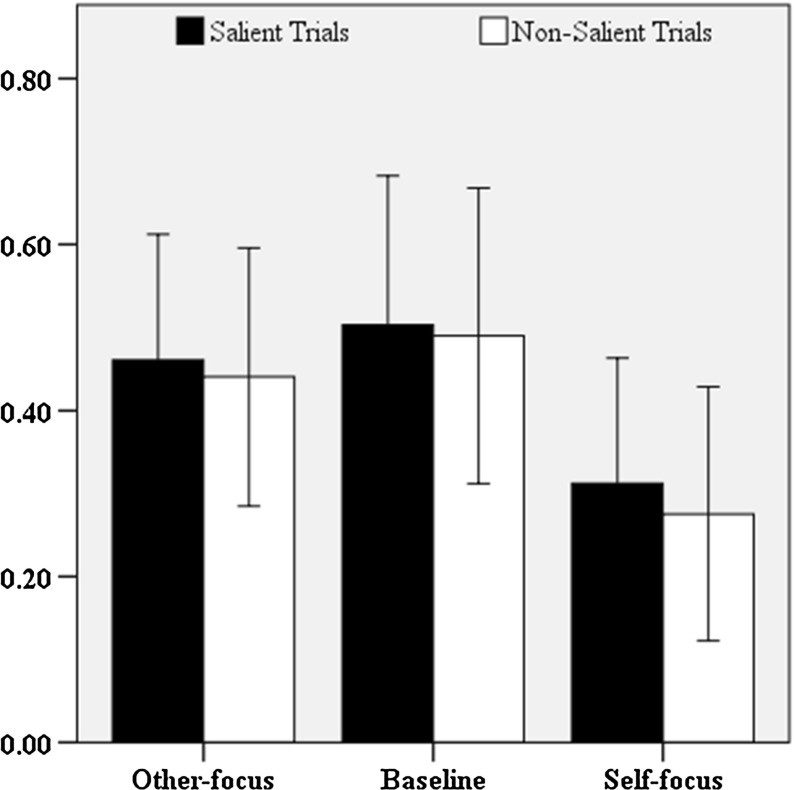
Mean proportions of speakers’ RPI for salient and non-salient trials. Error bars represent 95% confidence intervals

All dyads obtained the maximum of 40 points, indicating that they were able to correctly identify all targets. Per communicative setting, speakers provided 1200 object references (30 speakers * 40 trials). Out of the total of produced references (*n* = 3600), 10 (*n*_*baseline*_ = 2, *n*_*other*-*focus*_ = 5, *n*_*self*-*focus*_ = 3) were excluded due to errors in the experimental procedure. Speakers’ references consisted of noun phrases that contained zero to three adjectives. To estimate the amount speakers referred to privileged information, we counted the adjectives that matched the contrast presented in the stimuli (*n* = 1486). In Fig. 2, the mean proportions of speakers’ informational leakage as a function of the perspective manipulation (baseline, other-focus, self-focus), and whether the target and speakers’ privileged figure were similarly (salient trials) or differently (non-salient trials) shaped are shown. Overall, speakers in the baseline setting referred to privileged information in half of the produced references (50%), followed by the other-focused (45%), and self-focused speakers (29%). Across the three communicative settings, speakers seem to have referred to privileged information to the same degree on salient (43%) and non-salient (40%) trials.

The influence of the perspective manipulation and the interplay with the salience of speakers’ privileged information on the probability of privileged information to be mentioned was analyzed using a generalized linear mixed model analysis with a binomial distribution. For this we used the GLMER function from the lme4 package in R (version 3.3.0; CRAN project; R Core Team 2017). In order to obtain all comparisons appertaining to our hypotheses, four models were constructed. All these models treated the baseline setting as the reference category to which the two perspective settings were contrasted, and each model’s reference category also contained a different level of the two within-factors trial type (salient, non-salient) and contrast type (color, size). We constructed maximal models that included a full random effect structure (Barr et al. 2013). This maximal model included the perspective manipulation (self-focus, other-focus, baseline), the salience of the trials (salient, non-salient), the contrast (color, size) presented in the trials, and the setting * type and setting * contrast interactions as fixed factors. We included random intercepts and slopes for both speakers and experimental trials. Information about the models’ reference categories, and fixed and random-effects structures is presented in Tables 1, 2, 3 and 4. We report the results from the first model containing the comparison and of the maximal random effects structure that first converged (Barr et al. 2013). We used a Bonferroni correction to correct for multiple comparisons. The probability distribution was set on binomial with a logit link function and we used parametric bootstrapping over 100 iterations to estimate the confidence intervals and *p*-values. When the maximal model did not converge, we excluded random slopes with the lowest variance until convergence was reached.

**Table 1:** Estimated Coefficients and standard errors for the mixed model (M1) fitted to speakers’ RPI scores, using the baseline setting, non-salient trials and size contrasts as reference categories

	*B*	*SE b*	*95% CI*
Intercept	− 13.93	1.74	− 15.38, − 8.56
Setting other-focus (non-salient, size)	0.80	2.07	− 3.02, 5.11
Setting self-focus (non-salient, size)	1.28	1.66	− 1.98, 4.52
Type (salient, size in baseline)	1.53	0.81	− 0.33, 2.86
Contrast (non-salient, color in baseline)	− 1.91	1.93	− 6.59, 0.97
Setting other-focus * type (salient in size)	− 0.88	0.90	− 2.61, 0.93
Setting self-focus * type (salient in size)	− 0.30	0.90	− 2.03, 1.51
Setting other-focus * contrast (color in non-salient)	0.64	3.45	− 5.32, 8.20
Setting self-focus * contrast (color in non-salient)	− 4.24	5.11	− 14.67, 5.37

A comparison with the intercept-only model proved that the inclusion of the by-participant random slope for contrast and the by-item random slopes for type in M1 was justified by the data, *χ*^2^(4) = 173.8, *p* < .001

**Table 2:** Estimated coefficients and standard errors for the mixed model (M2) Fitted to speakers’ RPI scores, using the baseline setting, non-salient trials and color contrasts as reference categories

	*B*	*SE b*	*95% CI*
Intercept	− 15.08	1.75	− 17.80, − 10.94
Setting other-focus (non-salient, color)	1.24	1.90	− 1.67, 5.76
Setting self-focus (non-salient, color)	− 3.31	4.41	− 12.06, 5.21
Type (salient, color in baseline)	0.46	2.04	− 3.05, 4.93
Contrast (non-salient, size in baseline)	1.67	2.36	− 2.23, 7.01
Setting other-focus * type (salient in color)	− 0.79	1.13	− 3.10, 1.32
Setting self-focus * type (salient in color)	− 0.24	1.16	− 2.58, 1.97
Setting other-focus * contrast (size in non-salient)	− 0.17	3.12	− 6.55, 5.66
Setting self-focus * contrast (size in non-salient)	4.79	4.81	− 4.41, 14.43

A comparison with the intercept-only model proved that the inclusion of the by-participant random slopes for type and contrast, and the by-item random slope for type in M2 was justified by the data, *χ*^2^(7) = 174.7, *p* < .001

**Table 3:** Estimated coefficients and standard errors for the mixed model (M3) fitted to speakers’ RPI scores, using the baseline setting, salient trials and size contrasts as reference categories

	*B*	*SE b*	*95% CI*
Intercept	− 12.40	1.78	− 14.24, − 7.27
Setting other-focus (salient, size)	− 0.07	1.61	− 2.92, 3.38
Setting self-focus (salient, size)	0.98	1.56	− 1.86, 4.25
Type (non-salient, size in baseline)	− 1.53	1.00	− 3.29, 0.65
Contrast (salient, color in baseline)	− 1.91	2.23	− 6.81, 1.95
Setting other-focus * type (non-salient in size)	0.88	1.05	− 1.33, 2.79
Setting self-focus * type (non-salient in size)	0.30	0.96	− 1.70, 2.08
Setting other-focus * contrast (color in salient)	0.64	2.71	− 4.33, 6.27
Setting self-focus * contrast (color in salient)	− 4.24	4.43	− 13.71, 3.64

A comparison with the intercept-only model proved that the inclusion of the by-participant random slope for contrast, and the by-item random slope for type in M3 was justified by the data, *χ*^2^(4) = 173.81, *p* < .001

**Table 4:** Estimated coefficients and standard errors for the mixed model (M4) fitted to speakers’ RPI scores, using the baseline setting, salient trials and color contrasts as reference categories

	*B*	*SE b*	*95% CI*
Intercept	− 14.31	1.49	− 16.20, − 10.34
Setting other-focus (salient, color)	0.57	2.36	− 3.38, 5.88
Setting self-focus (salient, color)	− 3.26	4.41	− 12.64, 4.67
Type (non-salient, color in baseline)	− 1.53	0.90	− 3.31, 0.23
Contrast (salient, size in baseline)	1.91	2.32	− 2.01, 7.08
Setting other-focus * type (non-salient in color)	0.88	0.87	− 0.78, 2.64
Setting self-focus * type (non-salient in color)	0.30	1.03	− 1.73, 2.32
Setting other-focus * contrast (size in salient)	− 0.64	3.12	− 7.02, 5.21
Setting self-focus * contrast (size in salient)	4.24	4.62	− 4.19, 13.94

A comparison with the intercept-only model proved that the inclusion of the by-participant random slope for contrast, and the by-item random slope for type in M4 was justified by the data, *χ*^2^(4) = 173.8, *p* < .001

#### Influence of Perspective on Speakers’ RPI

Speakers’ RPI in the self- and other-focused setting did not significantly differ from speakers’ RPI in the baseline setting. For non-salient size trials, speakers in the other-focused (*M* = .33, *SD* = .45, *b* = 0.80, *SE* = 2.07, BC 95% CI [− 3.02, 5.11]), and self-focused setting (*M* = .24, *SD* = .41, *b* = 1.28, *SE* = 1.66, BC 95% CI [− 1.98, 4.52]), were just as likely as the baseline speakers (*M* = .44, *SD* = .50) to refer to privileged information. The same held for non-salient color trials: other-focused (*M* = .55, *SD* = .47, *b* = 1.24, *SE* = 1.90, BC 95% CI [− 1.67, 5.76]), and self-focused speakers’ RPI (*M* = .31, *SD* = .43, *b* = − 3.31, *SE* = 4.41, BC 95% CI [− 12.06, 5.21]) did not significantly differ from the baseline (*M* = .54, *SD* = .50). This pattern also held for salient size trials: speakers’ RPI in the other—(*M* = .34, *SD* = .44, *b* = − 0.07, *SE* = 1.61, BC 95% CI [− 2.92, 3.38]), and self-focused setting (*M* = .27, *SD* = .41, *b* = 0.98, *SE* = 1.56, BC 95% CI [− 1.86, 4.25]) did not significantly differ from the baseline (*M* = .46, *SD* = .50). Finally, speakers’ RPI on salient color trials in the other—(*M* = .58, *SD* = .46, *b* = 0.57, *SE* = 2.36, BC 95% CI [− 3.38, 5.88]), and self-focused setting (*M* = .35, *SD* = .42, *b* = − 3.26, *SE* = 4.41, BC 95% CI [− 12.64, 4.67]) did also not significantly differ from the baseline (*M* = .55, *SD* = .49).

#### Influence of Salience on Speakers’ RPI

In the baseline setting, the salience of privileged information did not influence speakers’ RPI. Baseline speakers were just as likely to refer to privileged information on non-salient (*M* = .44, *SD* = .50) and salient (*M* = .46, *SD* = .50) size trials (*b* = 1.53, *SE* = 0.81, BC 95% CI [− 0.33, 2.86]), and on non-salient (*M* = .54, *SD* = .50) and salient (*M* = .55, *SD* = .49) color trials (*b* = 0.46, *SE* = 2.04, BC 95% CI [− 3.05, 4.93]).

Baseline speakers’ RPI was also not influenced by the contrast presented in the trials. Speakers were just as likely to refer to privileged information on non-salient size (*M* = .44, *SD* = .50) and non-salient color (*M* = .54, *SD* = .50) trials (*b* = − 1.91, *SE* = 1.93, BC 95% CI [− 6.59, 0.97]), as on salient size (*M* = .46, *SD* = .50) and salient color (*M* = .55, *SD* = .49) trials (*b* = − 1.91, *SE* = 2.23, BC 95% CI [− 6.81, 1.95]).

When the two perspective settings were contrasted to the baseline setting, no significant differences were found. For size contrasting trials, no significant differences were found between the non-salient trials in the baseline setting (*M* = .44, *SD* .50), and salient trials in the other-focused setting (*M* = .34, *SD* = .44; *b* = − 0.88, *SE* = 0.90, BC 95% CI [− 2.61, 0.93]), nor between non-salient trials in the baseline setting and salient trials in the self-focused setting (*M* = .27, *SD* = .41; *b* = − 0.30, *SE* = 0.90, BC 95% CI [− 2.03, 1.51]). The same held for color contrasting trials. There were no significant differences between non-salient trials in the baseline setting (*M* = .54, *SD* = .50), and salient trials in the other-focused (*M* = .58, *SD* = .46; *b* = − 0.79, *SE* = 1.13, BC 95% CI [− 3.10, 1.32]), or in the self-focused setting (*M* = .35, *SD* = .42; *b* = − 0.24, *SE* = 1.16, BC 95% CI [− 2.58, 1.97]).

In addition, the contrast presented in the trials did not influence the extent to which speakers’ RPI differed in the other-focused or in the self-focused settings from the baseline. For non-salient trials, differences between size contrasting trials in the baseline setting (*M* = .44, *SD* = .50) and color contrasting trials in the other-focused (*M* = .55, *SD* = .47; *b* = 0.64, *SE* = 3.45, BC 95% CI [− 5.32, 8.20]), or color contrasting trials in the self-focused setting (*M* = .31, *SD* = .43; *b* = − 4.24, *SE* = 5.11, BC 95% CI [− 14.67, 5.37]) were non-significant. For salient trials, differences between size contrasting trials in the baseline setting (*M* = .46, *SD* = .50) and color contrasting trials in the other-focused setting (*M* = .58, *SD* = .46; *b* = 0.64, *SE* = 2.71, BC 95% CI [− 4.33, 6.27]), or color contrasting trials in the self-focused setting (*M* = .35, *SD* = .42; *b* = − 4.24, *SE* = 4.43, BC 95% CI [− 13.71, 3.64]) were also non-significant.

#### Speakers’ Introspective Perspective-Taking

Exploratory analysis showed that speakers’ introspective perspective-taking tendency was significantly non-normal, *D*(89) = 0.18, *p* < .001. We therefore performed a non-parametric Kruskal–Wallis test which revealed that speakers’ introspective perspective-taking tendency significantly differed between settings, *H*(2) = 7.07, *p* = .029. Step-down follow-up analysis that looks for homogeneous subsets showed that the perspective-taking tendencies of the other-focused speakers (*MRank* = 40.03) and the speakers in the baseline setting (*MRank* = 38.98) were homogeneous, *H* = .017, *p* = .896. Hence, the perspective-taking tendency between the other-focused speakers and the speakers in the baseline did not seem to significantly differ. The perspective-taking tendency of self-focused speakers (*MRank* = 54.67) did not belong to this homogeneous subset (*p* < .05). It therefore seems that self-focused speakers (*M* = 7.73, *SD* = 2.94) reported a significant higher perspective-taking tendency than the baseline speakers (*M* = 5.60, *SD* = 3.51) and the other-focused speakers (*M* = 5.62, *SD* = 3.63). To investigate whether speakers’ introspective perspective-taking tendency corresponded to their actual behavior during the game, a follow-up logit mixed model analysis was conducted. The full model included speakers’ self-report as fixed effect, a random intercept for subjects and items, and by-subject and by-item random slopes for the effect of speakers’ self-report. The *p*-values were obtained using the Likelihood Ratio Test (LRT) in which we compared the full model with the intercept only model. The LRT revealed that speakers’ self-report was a significant predictor of their actual RPI, *χ*^2^(5) = 120.41, *p* < .001. As speakers’ introspective perspective-taking tendency increased, they were less likely to have leaked privileged information during the game, *b* = − 3.80, *SE* = 0.52, *p* < .001.

#### Addressees’ Perception of Speakers’ Redundancy

Addressees’ perception of speakers’ redundancy was significantly non-normal, *D*(89) = 0.34, *p* < .001. A Kruskal–Wallis test showed that addressees’ perception of speakers’ redundancy during the game differed between settings, *H*(2) = 6.81 *p* = .033. Step-down follow-up analysis showed two homogeneous subsets. First, addressees’ perception of speakers’ redundancy tended to be homogeneous in the baseline (*MRank* = 36.50, *M* = 1.60, *SD* = 1.57) and in the self-focused setting (*MRank* = 47.30, *M* = 2.93, *SD* = 2.80), *H* = 3.83, *p* = .050. Moreover, these perception scores were homogeneous in the self-focused and in the other-focused setting (*MRank* = 51.41, *M* = 3.55, *SD* = 3.36), *H* = 0.48, *p* = .490. Addressees’ perception of speakers’ redundancy in the other-focused setting did not form a homogeneous subset with this perception score in the baseline. Since these perception scores were not equivalent, addressees seem to have indicated that other-focused speakers had provided them with significantly more redundant information than the speakers in the baseline.

## Intermediate Discussion

This first experiment examined whether eliciting speakers’ self- versus other-focus would influence their subsequent reference production. We found that speakers in the other- and self-focused settings were just as likely to refer to privileged information as the speakers whose perspective-taking was not manipulated. We did not replicate the results of Kaland et al. (2011) and Wardlow Lane et al. (2006) as the speakers in our study were just as likely to refer to private information, regardless of its salience.

An interesting finding of this study is the result of speakers’ introspective perspective-taking tendency and its relation to their reference production. Ironically, speakers with an elicited self-focus reported to have regarded their addressees’ perspective more than the speakers in the other two settings. These self-reported tendencies correlated negatively with speakers’ previous leakage behavior, indicating that speakers with a self-reported high perspective-taking tendency were less likely to have leaked private information during the game. In addition, speakers’ higher informational leakage in the other-focused setting was detected by the addressees who reported to have experienced more redundancy in the other-focused setting than in the baseline setting. It thus seems that not an elicited other- but *self*-focus activated speakers’ awareness of their interlocutor’s informational need, reducing the likelihood of speakers’ referring to privileged information. In a second experiment, we examine whether the explicit self-focus instructions could have made speakers’ more self-aware about their referential behavior than the speakers who were explicitly instructed to focus on their addressees’ perspective (Wicklund 1975), reducing the extent to which the self-focused speakers leak information that is privileged to them.

One limitation of the previous experiment we want to address is the fact that the majority of speakers (65.56%) persisted in a certain reference strategy throughout the experiment. That is, 59 speakers either referred to color and size contrasts on all of the trials, or they refrained from including any adjectives at all throughout the game. This tendency to retain a certain reference strategy could have interfered with the speakers’ audience design (Horton and Gerrig 2002), and thus to the extent to which they were influenced by the elicited perspective and the salience of their privileged knowledge.

One factor that could have contributed to that speakers’ consistency is the self-paced method by which speakers completed the trials. In a self-paced manner, speakers clicked on their private computerscreen to receive the ‘occlude’ and ‘identify’ instructions and the perspective manipulation. This self-paced method could have resulted in a routine by which speakers performed the instructions and completed the trials. Moreover, the fact that the perspective-taking manipulation appeared on speakers’ private computer screen—in which it was not made visible which figures were commonly versus privately known—could have reduced the intrusiveness of the perspective-taking manipulation. Although speakers were explicitly trained to return their attention from their private screen to the physical context shared between them and their addressee *before* they identified the target figure, the possibility exists that speakers were still regarding their private screen (in which perspectives were not marked) while formulating their reference. This raises the question whether speakers were actually regarding the common-ground status before they referred to the target figure. In a second experiment, we address this issue by asking speakers explicitly to indicate on the common-ground cards before them and their addressee which figures were visible to either the speakers themselves or to their addressee.

Another possible explanation as to why speakers maintained a consistent reference strategy is the fact that the six color manipulations used in the experimental design (following Kaland et al. 2011, 2014) could have inspired speakers to refer to color contrasts on all trials (e.g., Koolen et al. 2013). In a second experiment, we address this issue by reducing the obtrusiveness of the color manipulation.

## Experiment 2

The second experiment addresses the issue of speakers’ retained reference strategy by amplifying the intrusiveness of the perspective-taking manipulation, and reducing the obtrusiveness of the color-attributes. Recall that the first experiment exposed speakers to the perspective-taking manipulation on their private computerscreen. On this screen, speakers indicated which figures were either visible to their addressee or to themselves. Differences in perspectives were not visibly marked on this screen, but they were visibly marked on the cards lying on the table in between speakers and addressees. To guarantee that speakers are explicitly aware of the common-ground status before they produce a referential message, we asked our speakers in the second experiment to use the four figures lying in between them and their addressee to indicate which ones are either visible to their addressee (other-focus) or to themselves (self-focus). Moreover, instead of employing the six color versus the two size manipulations, the second study reduces the color manipulations from six to two (blue, green), thereby equalizing the amount of color manipulations to the two size manipulations (big, small) employed. With this more robust design, we test the hypotheses that, compared to the baseline, other-focused speakers will be less likely and self-focused speakers will be more likely to refer to privileged information. Furthermore, we explore the assumption that speakers will be more likely to leak information privileged to them when this information is salient rather than non-salient, and test the hypothesis that the salience of speakers’ privileged information will interact with the explicit perspective-focus (other-focus, self-focus). In addition, we address the finding from the previous experiment that the self-focused speakers in the first experiment seem to have been, ironically, more aware of the required audience design than the other-focused speakers or the speakers partaking in the baseline setting. Previous studies indicated that strengthening people’s attention to the self can reduce egocentric behavior (Hass 1984; Stephenson and Wicklund 1984). According to the objective self-awareness theory (Wicklund 1975), persons who are privately self-aware act on their cognitions that are salient at that specific moment in time, whereas publicly self-aware (Fenigstein 1987; Govern and Marsch 2001) persons reflect on themselves as if they are an object under scrutinization. Under this scrutinization, the difference between their actual and required behavior, derived from the standards that apply to the interaction, becomes salient. Our self-focused speakers could have found themselves in such a reflective state, especially since a cue of their addressees’ different perspective was present (Gendolla and Wicklund 2009). Speakers were able to see which figures were available for addressees’ selection process (and which one was not). A higher self-awareness in the self-focused rather than other-focused speakers could have caused the self-focused speakers to engage in a more accurate audience design, thereby being less likely to leak privileged information. Hence, in addition to our hypotheses from experiment 1, we explore the possibility that (a) public self-awareness will be higher for speakers focusing on their own perspective (i.e., self-focused speakers) than those focusing on their addressees’ point of view (i.e., other-focused speakers) or those speakers without an induced perspective (i.e., baseline), and (b) public self-awareness will influence the extent to which speakers oblige with the salient social standard (i.e., engaging in an accurate audience design), expressed by the reduced probability of speakers leaking information about their egocentric perspective.

### Method

#### Participants

114 student-dyads (*n* = 228) participated in the second experiment. None of the participants had participated in the first experiment. The data of 11 dyads were excluded from analyses, due to an error in the experimental procedure (*n* = 22). The analyses were based on 103 dyads in which the participants were randomly assigned either the role of the speaker (69 women, 34 men, *M*_*age*_ = 21.8 years; age range 17–52 years) or the role of the addressee (74 women, 29 men, *M*_*age*_ = 21.7 years; age range 17–47). All participants were fluent in Dutch, did not experience problems at discerning the colors used in the study, and received course credits for their participation.

#### Design, Materials and Procedure

Following the first experiment, speakers were randomly allocated to one of the experimental settings (self-focus, other-focus, baseline). This resulted in 33 dyads in the self-focused setting, 35 dyads in the other-focused setting, and 35 in the baseline setting. We replicated the design, materials and procedure from the first experiment, with the exceptions that (a) the experiment was now paced by the experimental leader, and (b) the number of colors used was reduced from six to two (blue, green). To ensure the salience of the size contrasts used, we amplified the size difference between the target and speakers’ privileged figure from 1:2 (experiment 1) to 1:4.

##### Eliciting Self-Versus Other-Focus

**Fig. 3:**
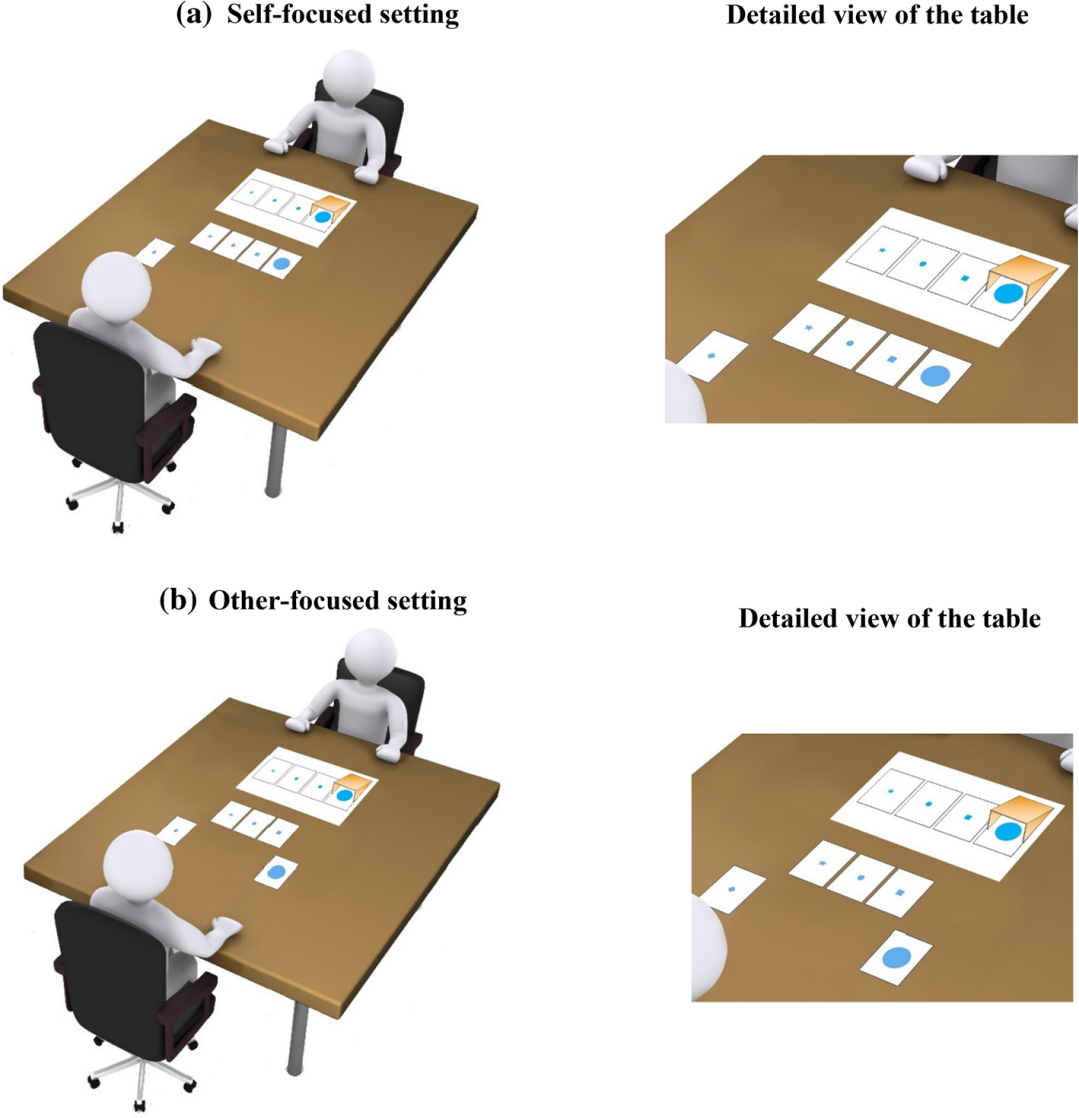
The perspective-taking manipulation in the self-focused (**a**) and other-focused (**b**) setting

The computerscreen was omitted from the experimental procedure and all instructions took place on the table in front of the speakers and addressees. The experimental leader made sure that addressees kept their eye shut during the entire procedure. After speakers had blocked one figure from their addressees’ view, speakers in the two perspective settings were provided with five carbon cards depicting the four figures lying on the table in front of them and one filler figure. The experimental leader asked speakers in the self-focused setting to indicate *which four figures were visible to them* by moving the intended four figures upwards (Fig. 3a). The experimental leader would then check speakers’ selection and confirm speakers’ egocentric perspective by turning the filler figure—so that the blank side of the card was visible—and by stressing that the four selected cards were indeed the four figures speakers were seeing. In the other-focused setting, the experimental leader asked speakers to move upward *the three figures that were visible to [name of the addressee]*, eliciting addressees’ perspective of the situation. In contrast to experiment 1, the other-focus prompt included the name of the addressee to enhance speakers’ focus to their addressees’ perspective. The selected figures were checked by the experimental leader and, as in the self-focused setting, she would turn speakers’ privileged figure and the filler after which she would confirm speakers’ selection by stressing that the three figures moved upwards were indeed the ones the addressee was seeing (Fig. 3b). As in the first experiment, speakers in the baseline setting were not confronted with perspective questions. As such, these speakers’ self- or other-focus was not reinforced.

##### Speakers’ Introspective Perspective-Taking

Note that in the first experiment speakers’ introspective perspective-taking was measured by only one item on a 10-point scale (i.e., “*To what extent did you take into account your addressee’s perspective during the game?*”). To increase the validity of the self-reports, we measured speakers’ perspective-taking tendency by a multiple-item scale, adjusting the perspective-taking items presented in Davis’ Interpersonal Reactivity Index (IRI; 1980) to the referential communication setting. Our introspective perspective-taking scale measured speakers’ consciousness about the difference in perspectives presented to them (e.g., “*I was aware that the addressee had a different view of the situation than I*”), and speakers’ acknowledgement of these differences in perspectives expressed by their (adjusted) referential communication (e.g., “*I adjusted my instructions to the informational need of the addressee*”). The eleven introspective perspective-taking questions were alternated by fourteen filler questions (e.g., “*Playing the game made me enthusiastic*”). Speakers’ answered the declarative sentences on a 7-point scale (1 = *totally disagree*, 7 = *totally agree*). The introspective perspective-taking scale had a high reliability (Cronbach’s α = .89). The individual questions are presented in Table 5.

**Table 5:** Items of speakers’ introspective perspective-taking scale

Item
1. During the game, I was especially aware of the figures that were visible to me (R)
2. During the game, I found it difficult to put myself in the addressee’s position (R)
3. I was aware that the addressee had a different view of the situation than I
4. During the game, I was especially aware of the figures I was seeing (R)
5. During the game, I mainly took into account my own view of the situation (R)
6. I played the game as much as possible from addressee’s point of view
7. Before I gave a description, I tried to imagine the situation from the addressee’s perspective
8. Placing myself in the addressee’s position allowed me to take into account which information the addressee needed in order to select the intended figure
9. I have described the figures with just enough (not too much, not too little) information
10. The addressee did not need much of the information provided by me to select the intended figure (R)
11. During the game, I adapted my descriptions to addressee’s informational need (i.e., the information the addressee needed to select the intended figure) as much as possible

The (R) signals that the scores were recoded before the analysis

##### Speakers’ Situational Self-Awareness

To explore the possibility that an elicited self-focus enhanced speakers’ public self-awareness, speakers filled out a Dutch translated version of the Situational Self-Awareness Scale (SSAS; Govern and Marsch 2001). The SSAS measures two dimensions of speakers’ self-awareness, namely their private and their public self-awareness. According to the objective self-awareness theory (Wicklund 1975), privately self-aware persons will act on salient inner cognitions, whereas publicly self-aware persons will modify their behavior so that it adheres to the social standard in order to prevent a negative evaluation (Froming et al. 1982; Gendolla and Wicklund 2009). The scale also takes into account the non-self-aware individuals by measuring the extent to which these persons are focused on their surroundings. This resulted in a three-factor scale (Surroundings, Private, Public), and each factor was measured by three items phrased as declarative sentences (e.g., “*Right now*, *I am conscious of my inner feelings*”). Speakers responded how much they agreed with the sentences on a 7-point scale (1 = *strongly disagree*; 7 = *strongly agree*). The items, their factor loadings and Cronbach’s Alpha’s are presented in Table 6.

**Table 6:** Items of the situational self-awareness scale (Govern and Marsch 2001)

Item	Factor loading			Cronbach’s Alpha	
	Factor	Govern and Marsch (2001)	Experiment 2	If item deleted	Based on all items
1. Right now, I am keenly aware of everything in my environment	Surroundings	.81	.92	.78	.85
2. Right now, I am conscious of my inner feelings	Private	.68	.69	.72	.74
3. Right now, I am concerned about the way I present myself	Public	.80	.83	.81	.86
4. Right now, I am self-conscious about the way I look	Public	.88	.87	.76	.86
5. Right now, I am conscious of what is going on around me	Surroundings	.75	.73	.83	.85
6. Right now, I am reflective about my life	Private	.76	.64	.74	.74
7. Right now, I am concerned about what other people think of me	Public	.85	.83	.82	.86
8. Right now, I am aware of my innermost thoughts	Private	.84	.89	.46	.74
9. Right now, I am conscious of all objects around me	Surroundings	.78	.84	.76	.85

Item 6 loaded .55 on factor public and item 2 loaded .41 on factor surroundings. All other items loaded below .40 on the other two factors in the scale

##### Addressees’ Perception of Speakers’ Perspective-Taking

The eleven items from the speakers’ introspective perspective-taking scale described above were reformulated into addressees’ perspective. In this way, addressees reported their perception of speakers’ perspective-taking behavior during the referential communication game. The reformulated items are presented in Table 7. Addressees’ version of the scale had a high reliability (Cronbach’s α = .78).

**Table 7:** Items of addressees’ perception of speakers’ perspective-taking scale

Item
1. During the game, the speaker mainly took into account his or her own view of the situation (R)
2. The speaker described the figures with just enough (not too much, not too little) information
3. The speaker played the game as much as possible from my point of view
4. Before the speaker gave a description, (s)he tried to imagine the situation from my perspective
5. I did not need much of the information that was provided by the speaker to select the intended figure (R)
6. During the game, the speaker was especially aware of the figures (s)he was seeing (R)
7. During the game, the speaker found it difficult to place him or herself in my position (R)
8. During the game, the speaker adapted his or her descriptions to my informational need (i.e., the information I needed to select the intended figure) as much as possible
9. The speaker was aware that I had a different view of the situation than he or she did
10. During the game, the speaker was especially aware of the figures that were visible to him or herself (R)
11. By placing him or herself in my position allowed the speaker to take into account which information I needed in order to select the intended figure

The (R) signals that the scores were recoded before the analysis

### Results

**Fig. 4:**
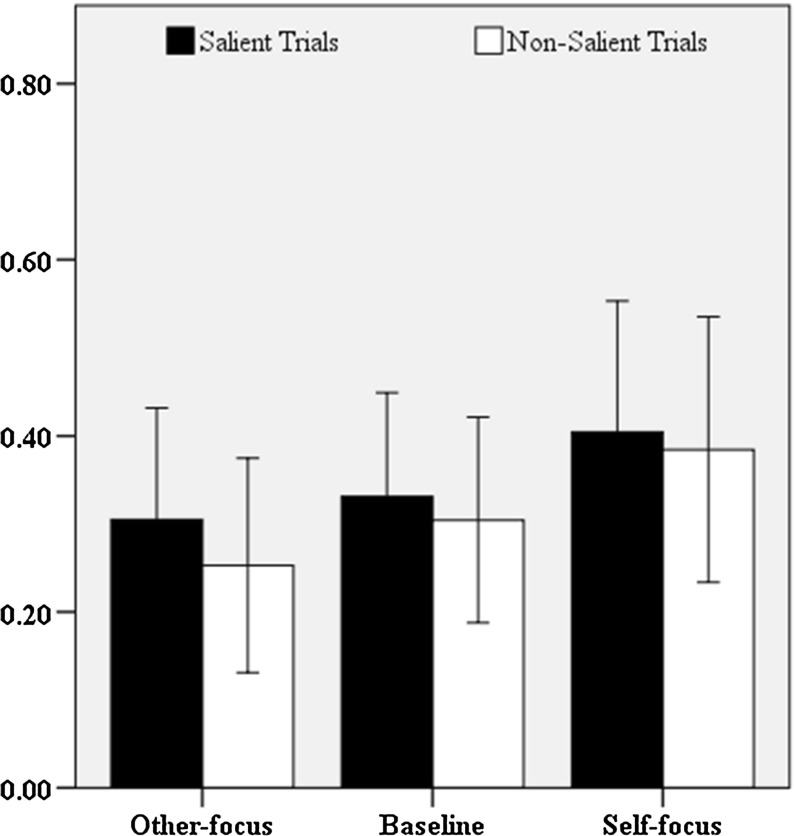
Mean proportions of speakers’ RPI. Error bars represent 95% confidence intervals

All dyads were able to correctly identify the forty targets. Out of the total of produced references (*n* = 4160), 15 references (*n*_*baseline*_ = 2, *n*_*other*-*focus*_ = 4, *n*_*self*-*focus*_ = 9) were excluded due to errors in the experimental procedure. As in the previous experiment, we counted the adjectives that leaked information about speakers’ privileged perspective (*n* = 1353). In Fig. 4, the mean proportions of speakers’ informational leakage as a function of the perspective manipulation (baseline, other-focus, self-focus), and whether the target and speakers’ privileged figure were similarly (salient trials) or differently (non-salient trials) shaped are presented. Overall, speakers in the self-focused setting referred to privileged information in 39% of the produced references, followed by speakers in the baseline setting (32%), and speakers in the other-focused setting (28%). As in experiment 1, speakers across the three perspective settings seem to have referred to privileged information to the same degree for salient (35%) and non-salient (31%) trials.

We replicated the method of analysis from the previous experiment, and we excluded random slopes to reduce multicollinearity (< 3). The models’ fixed and random effects structures, beta coefficients and confidence intervals are presented in Tables 8, 9, 10 and 11.

**Table 8:** Estimated coefficients and standard errors for the mixed model (M5) fitted to speakers’ RPI scores, using the baseline setting, non-salient trials and size contrasts as reference categories

	*B*	*SE b*	*95% CI*
Intercept	− 9.60	2.86	− 13.84, − 2.63
Setting other-focus (non-salient, size)	2.49	2.94	− 2.74, 8.79
Setting self-focus (non-salient, size)	5.02	3.07	− 1.36, 10.69
Type (salient, size in baseline)	0.72	0.51	− 0.34, 1.68
**Contrast (non-salient, color in baseline)**	**7.72**	**3.11**	**2.00, 14.20**
Setting other-focus * type (salient in size)	0.53	0.44	− 0.29, 1.42
Setting self-focus * type (salient in size)	− 0.09	0.38	− 0.78, 0.71
Setting other-focus * contrast (color in non-salient)	− 3.68	2.56	− 8.80, 1.24
Setting self-focus * contrast (color in non-salient)	− 2.43	3.26	− 9.74, 3.05

Significant results are represented in bold. A comparison with the intercept-only model proved that the inclusion of the by-participant random slope for contrast and the by-item random slopes for setting and type in M5 was justified by the data, *χ*^2^(11) = 402.80, *p* < .001

**Table 9:** Estimated coefficients and standard errors for the mixed model (M6) fitted to speakers’ RPI scores, using the baseline setting, non-salient trials and color contrasts as reference categories

	*B*	*SE b*	*95% CI*
Intercept	− 1.89	4.55	− 9.06, 8.79
Setting other-focus (non-salient, color)	− 1.17	4.57	− 8.93, 8.97
Setting self-focus (non-salient, color)	2.61	4.91	− 8.71, 10.53
Type (salient, color in baseline)	0.61	0.70	− 0.78, 1.98
**Contrast (non-salient, size in baseline)**	− **7.78**	**3.34**	− **14.56,** − **1.48**
Setting other-focus * type (salient in color)	0.57	0.58	− 0.62, 1.67
Setting self-focus * type (salient in color)	0.03	0.54	− 1.10, 1.02
Setting other-focus * contrast (size in non-salient)	3.68	3.02	− 2.53, 9.31
Setting self-focus * contrast (size in non-salient)	2.43	3.33	− 2.78, 10.25

Significant results are represented in bold. A comparison with the intercept-only model proved that the inclusion of the by-participant random slopes for type and contrast, and the by-item random slopes for setting and type in M6 was justified by the data, *χ*^2^(14) = 404.97, *p* < .001

**Table 10:** Estimated coefficients and standard errors for the mixed model (M7) fitted to speakers’ RPI scores, using the baseline setting, salient trials and size contrasts as reference categories

	*B*	*SE b*	*95% CI*
Intercept	− 9.06	3.49	− 13.71, − 0.02
Setting other-focus (salient, size)	3.09	− 0.27	− 2.36, 9.08
Setting self-focus (salient, size)	5.07	3.53	− 2.14, 11.68
Type (non-salient, size in baseline)	− 0.61	0.77	− 2.22, 0.79
**Contrast (salient, color in baseline)**	**7.78**	**2.96**	**2.59, 14.20**
Setting other-focus * type (non-salient in size)	− 0.57	0.54	− 1.65, 0.46
Setting self-focus * type (non-salient in size)	− 0.03	0.63	− 1.27, 1.19
Setting other-focus * contrast (color in salient)	− 3.68	2.40	− 8.12, 1.29
Setting self-focus * contrast (color in salient)	− 2.43	2.80	− 8.83, 2.15

Significant results are represented in bold. A comparison with the intercept-only model proved that the inclusion of the by-participant random slopes for type and contrast, and the by-item random slopes for setting and type in M7 was justified by the data, *χ*^2^(14) = 404.97, *p* < .000

**Table 11:** Estimated coefficients and standard errors for the mixed model (M8) fitted to speakers’ RPI scores, using the baseline setting, salient trials and color contrasts as reference categories

	*B*	*SE b*	*95% CI*
Intercept	− 0.67	3.44	− 6.01, 7.46
Setting other-focus (salient, color)	− 0.18	3.21	− 6.16, 6.44
Setting self-focus (salient, color)	1.41	3.77	− 6.56, 8.22
Type (non-salient, color in baseline)	− 1.08	1.29	− 3.16, 1.91
**Contrast (salient, size in baseline)**	− **8.02**	**2.65**	− **13.40,** − **3.01**
Setting other-focus * type (non-salient in color)	− 0.39	0.91	− 2.20, 1.35
Setting self-focus * type (non-salient in color)	0.66	1.00	− 1.62, 2.30
Setting other-focus * contrast (size in salient)	3.57	2.45	− 1.00, 8.61
Setting self-focus * contrast (size in salient)	2.87	2.76	− 1.95, 8.86

Significant results are represented in bold. A comparison with the intercept-only model proved that the inclusion of the by-participant random slopes for type * contrast, and the by-item random slopes for setting and type in M8 was justified by the data, *χ*^2^(18) = 416.42, *p* < .000

#### Influence of Perspective on Speakers’ RPI

Speakers’ RPI in the self- and other-focused setting did not significantly differ from speakers’ RPI in the baseline setting. For non-salient size trials, speakers in the other-focused (*M* = .22, *SD* = .37, *b* = 2.49, *SE* = 2.94, BC 95% CI [− 2.74, 8.79]), and self-focused setting (*M* = .31, *SD* = .43, *b* = 5.02, *SE* = 3.07, BC 95% CI [− 1.36, 10.69]), were just as likely as the baseline speakers (*M* = .15, *SD* = .32) to refer to privileged information. The same held for non-salient color trials: other-focused (*M* = .29, *SD* = .41, *b* = − 1.17, *SE* = 4.57, BC 95% CI [− 8.93, 8.97]), and self-focused speakers’ RPI (*M* = .45, *SD* = .47, *b* = 2.61, *SE* = 4.91, BC 95% CI [− 8.71, 10.53]) did not significantly differ from the baseline (*M* = .45, *SD* = .47). This pattern also held for salient size trials: speakers’ RPI in the other—(*M* = .25, *SD* = .40, *b* = 3.09, *SE* = − 0.27, BC 95% CI [− 2.36, 9.08]), and self-focused setting (*M* = .32, *SD* = .44, *b* = 5.07, *SE* = 3.53, BC 95% CI [− 2.14, 11.68]) did not significantly differ from the baseline (*M* = .17, *SD* = .33). Finally, speakers’ RPI on salient color trials in the other—(*M* = .36, *SD* = .40, *b* = − 0.18, *SE* = 3.21, BC 95% CI [− 6.16, 6.44]), and self-focused setting (*M* = .49, *SD* = .45, *b* = 1.41, *SE* = 3.77, BC 95% CI [− 6.56, 8.22]) did also not significantly differ from the baseline (*M* = .49, *SD* = .45).

#### Influence of Salience on Speakers’ RPI

Baseline speakers’ RPI was influenced by the contrast presented in the trials. Speakers were more likely to refer to privileged information on non-salient color trials (*M* = .45, *SD* = .47) than on non-salient size (*M* = .15, *SD* = .32) trials (*b* = 7.72, *SE* = 3.11, BC 95% CI [2.00, 14.20]). This pattern also held for salient trials as speakers were more likely to leak information on salient trials depicting a contrast in color (*M* = .49, *SD* = .45) than on trials depicting a contrast in size (*M* = .17, *SD* = .33) trials (*b* = 7.78, *SE* = 2.96, BC 95% CI [2.59, 14.20]).

However, the salience of privileged information did not influence baseline speakers’ RPI. Baseline speakers were just as likely to refer to privileged information on non-salient (*M* = .15, *SD* = .32) and salient (*M* = .17, *SD* = .33) size trials (*b* = 0.72, *SE* = 0.51, BC 95% CI [− 0.34, 1.68]), and on non-salient (*M* = .45, *SD* = .47) and salient (*M* = .49, *SD* = .45) color trials (*b* = 0.61, *SE* = 0.70, BC 95% CI [− 0.78, 1.98]).

The salience of the trials did not influence the difference in speakers’ RPI between the baseline and other-focused setting, and the baseline and self-focused setting. For size contrasting trials, no significant differences were found between non-salient trials in the baseline setting (*M* = .15, *SD* = .32) and salient trials in the other-focused setting (*M* = .25, *SD* = .40), *b* = 0.53, *SE* = 0.44, BC 95% CI [− 0.29, 1.42], nor between non-salient trials in the baseline setting and salient trials in the self-focused setting (*M* = .32, *SD* = .44), *b* = − 0.09, *SE* = 0.38, BC 95% CI [− 0.78, 0.71]. The same held for color contrasting trials, as there were no significant differences between non-salient trials in the baseline setting (*M* = .45, *SD* = .47) and salient trials in the other-focused (*M* = .36, *SD* = .40), *b* = 0.57, *SE* = 0.58, BC 95% CI [− 0.62, 1.67], or self-focused (*M* = .49, *SD* = .45) setting, *b* = 0.03, *SE* = 0.54, BC 95% CI [− 1.10, 1.02].

Further, the contrast presented in the trials did not influence the extent to which speakers’ RPI differed between the baseline and other-focused setting, nor between the baseline and the self-focused setting. For non-salient trials, differences between size contrasting trials in the baseline setting (*M* = .15, *SD* = .32) and color contrasting trials in the other-focused setting (*M* = .29, *SD* = .41), *b* = − 3.68, *SE* = 2.56, BC 95% CI [− 8.80, 1.24], and color contrasting trials in the self-focused setting (*M* = .45, *SD* = .47), *b* = − 2.43, *SE* = 3.26, BC 95% CI [− 9.74, 3.05], were not significant. In addition, for salient trials, differences between size contrasting trials in the baseline setting (*M* = .17, *SD* .33) and color contrasting trials in the other-focused setting (*M* = .36. *SD* = .40), *b* = − 3.68, *SE* = 2.40, BC 95% CI [− 8.12, 1.29], and self-focused setting (*M* = .49, *SD* = .45), *b* = − 2.43, *SE* = 2.80, BC 95% CI [− 8.83, 2.15], were non-significant.

#### Speakers’ Introspective Perspective-Taking

Speakers’ self-reported perspective-taking tendency was significantly non-normal, *D*(103) = 0.11, *p* < .01. A Kurskal-Wallis analysis revealed that speakers’ introspective perspective-taking tendency did not significantly differ between settings, *H*(2) = 0.28, *p* = .854. Speakers in the baseline (*M* = 5.17, *SD* = 1.02), other-focused (*M* = 5.08, *SD* = 1.31), and self-focused setting (*M* = 4.95, *SD* = 1.29) reported to have engaged in perspective-taking behavior to the same degree. As in the first study, we conducted a follow-up logit mixed model analysis to investigate whether speakers’ introspective perspective-taking tendency corresponded with their tendency to refer to privileged information (RPI) during the game. This (full) model included speakers’ self-report as fixed effect, by-subject and by-item random intercepts, and a by-subject random slope for speakers’ self-report. LRT Model comparison revealed that a by-item random slope for speakers’ self-report did not increase the model’s fit, *χ*^2^ (3) = 0.33, *p* = .847. LRT comparison between the intercept only and the full model revealed that speakers’ self-report was a significant predictor of their RPI during the game, *χ*^2^ (3) = 29.63, *p* < .001. As speakers’ perspective-taking tendency increased, they were less likely to have leaked privileged information during the game, *b* = − 2.16, *SE* = 0.48, *p* < .001.

#### Speakers’ Situational Self-Awareness

**Fig. 5:**
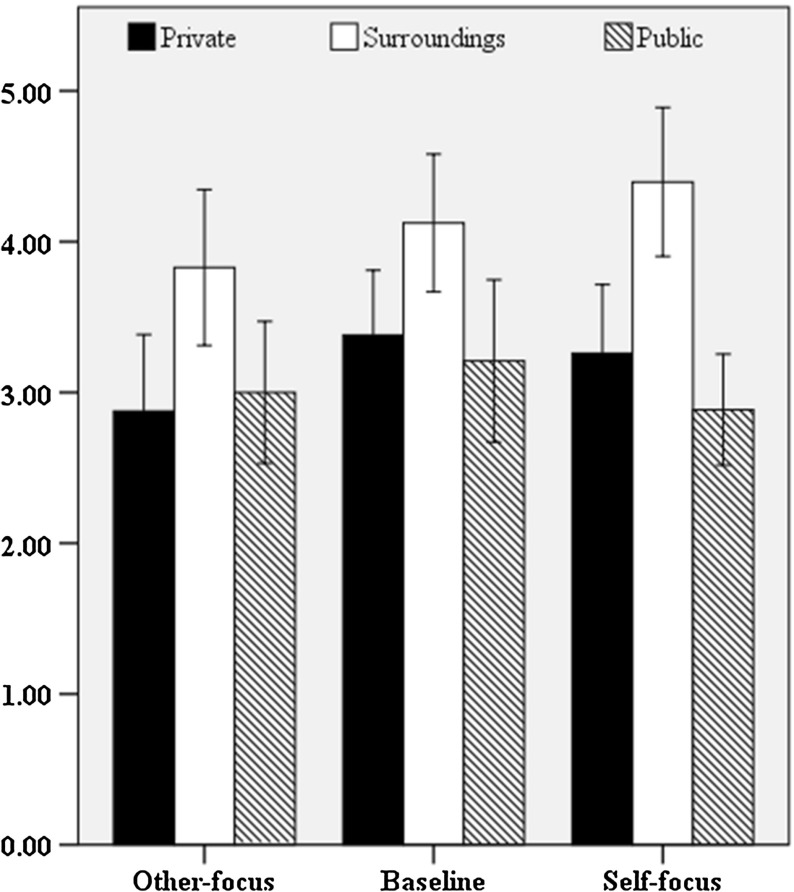
Means of speakers’ self-awareness (private, surroundings, public) in the other-focused, baseline and self-focused settings. Error bars represent 95% confidence intervals

We investigated the extent to which the perspective manipulation (other-focus, self-focus, baseline) would influence speakers’ amount of experienced non-(surroundings), public and private self-awareness. The self-awareness scores per communicative setting are presented in Fig. 5.

Exploratory analyses revealed that we had to exclude the data of one participant in the self-focused setting who appeared to be an outlier. Normality slightly improved, but the public self-awareness scores remained significantly non-normal in the baseline, *D* (35) = 0.13, *p* = .029, and in the other-focused setting, *D* (35) = 0.17, *p* = .006, as the private self-awareness score in the other-focused setting, *D* (35) = 0.16, *p* = 0.16. For public (*z* = 2.61, *p* < .05) and private (*z* = 1.70, *p* > .05) self-awareness, the data were slightly positively skewed. We employed a Kruskal–Wallis test on the data of the remaining 102 participants (*n*_*baseline*_ = 35, *n*_*other*-*focus*_ = 35, *n*_*self*-*focus*_ = 32). Findings showed that perspective-focus did not have a significant effect on speakers’ amount of public self-awareness, *H*(2) = 0.22, *p* = .897, surroundings, *H*(2) = 2.35, *p* = .309, and private self-awareness, *H*(2) = 3.82, *p* = .148. Contrary to our expectations, self-focused speakers (*M* = 2.89, *SD* = 1.02) were not publicly more self-aware than other-focused speakers (*M* = 3.00, *SD* = 1.37) or the speakers partaking in the baseline setting (*M* = 3.21, *SD* = 1.57). In addition, no differences in the extent to which speakers were privately self-aware were found between the other-focused (*M* = 2.88, *SD* = 1.48), self-focused (*M* = 3.26, *SD* 1.26), and baseline (*M* = 3.26, *SD* = 1.26) settings. This also held for the extent to which speakers were focused on their surroundings between the baseline (*M* = 4.12, *SD* = 1.33), other-focused (*M* = 3.83, *SD* = 1.50), and self-focused (*M* = 4.40, *SD* = 1.37) setting.

A follow-up logit mixed model analyses was conducted to test the hypothesis that public self-awareness would influence the extent to which speakers would oblige with the required audience design, expressed by less informational leakage. The full model included speakers’ public self-awareness as fixed effect, and by-subject and by-item random intercepts. Model comparison (LRT) revealed that by-subject and by-item random slopes for public did not increase the model’s fit, *χ*^2^(4) = 0.33, *p* = .988. Comparison between the intercept only and the full model indicated that speakers’ amount of public self-awareness was not a significant predictor of informational leakage during the game, *χ*^2^(1) = 0.16, *p* = .685, *b* = 0.16, *SE* = 0.39.

#### Addressees’ Perception of Speakers’ Perspective-Taking

Addressees’ perception of speakers’ perspective-taking was normally distributed, *D*(103) = 0.07, *p* = .200. Addressees in the baseline (*M* = 5.17, *SD* = 0.98), other-focus (*M* = 5.21, *SD* = 0.87), and self-focus (*M* = 5.29, *SD* = 0.77) setting perceived speakers’ perspective-taking tendency to the same degree, *F*(2, 100) = 0.16, *p* = .850.

## Discussion

With a more robust experimental design, we examined the influence of explicit perspective-taking instructions on speakers’ tendency to leak information privileged to them. In our previous experiment, factors such as the experimental procedure and design features could have influenced the extent to which speakers retained a certain reference strategy throughout the game, interfering with the perspective-taking manipulation. By addressing these factors, we were able to successfully reduce the number of speakers routinely persisting in using a certain reference strategy from 59 (65.56%) in the first experiment to 44 speakers (42.72%) in the second one. We replicated the results of the first experiment by showing that the perspective-manipulation did not influence speakers’ reference production. Other-focused and self-focused speakers were just as likely to refer to private information as the speakers who were not confronted with an explicit perspective-focus. The second experiment did also show that speakers were just as likely to refer to information not known to the addressee, regardless whether this privileged information was salient to them.

As in the first experiment, speakers’ introspective perspective-taking tendency predicted their reference production during the experiment. In particular, speakers who reported to have regarded their addressees’ perspective were less likely to have leaked information about their private knowledge to their addressee. However, these tendencies did not depend on the induced speakers’ perspective-focus. Whether speakers were explicitly focused on their own perspective or on the perspective of their addressee, they all reported to have regarded the addressees’ perspective to the same degree. Addressees also reported that speakers had taken their perspective into account, regardless of speakers’ stimulated perspective-focus (self-focus, other-focus, baseline). The results of speakers’ and addressees’ self-reports additionally suggest that the explicit self-versus other-focus did not influence speakers’ reference behavior.

In this second experiment, we also tested the hypothesis that an explicit self-focus, rather than an explicit other-focus, would make speakers’ more aware of their own reference production, and whether this awareness would result in less informational leakage. Results showed that speakers’ amount of experienced public, private or non-self-awareness did not differ across the communicative settings. In addition, whether speakers were publicly, privately or non-self-aware, speakers were just as likely to refer to information not known to their addressee.

## General Discussion

Two experiments investigated the question whether an explicit attention to addressees’ perspective influences speakers’ audience design during reference production. The intriguing finding of the research presented here is that making speakers explicitly aware of their addressees’ perspective did not appear to influence the extent to which they adjust their reference production to addressees’ knowledge and attentional status. Contrary to what theories of audience design suggest (Clark and Murphy 1982), speakers still leaked information privileged to them, regardless of their explicit awareness of addressees’ informational need. The results of the two experiments presented in this paper indicate the complex nature of perspective-taking. Even during an easy collaborative task in which speakers had enough cognitive resources left to engage in perspective-taking, explicit instructions to regard common-ground knowledge did not reduce speakers’ egocentrism.

That we evidenced speakers’ informational leakage in the baseline as well as in the perspective-taking (i.e., other-focus) setting seems to suggest that perspective-taking—in the form of an accurate audience design—is not necessarily incorporated in the planning of utterances during language production. In this sense, the findings support previous research claiming that speakers plan their referential utterances on the basis of the knowledge that is immediately available to themselves, regardless of whether this information is shared and commonly known between them and their partner (e.g., Barr and Keysar 2005; Epley et al. 2004). In these two experiments, perspective-taking did not constrain speakers’ reference production. In this sense, to take over the other’s perspective during conversation and using this information to construe a referential utterance that corresponded to the addressee’s perspective was optional instead of obligatory. By comparing speakers’ informational leakage in a baseline referential setting to a setting in which they were self- versus other-focused and finding that neither an induced self- nor other-focus influenced speakers’ informational leakage,^2^ we conclude that egocentrism prevailed. Even when speakers had an explicit knowledge of addressees’ perspective, they were just as likely to refer to their egocentric knowledge, disregarding the perspective of their addressee.

We have two reasons to believe that the speakers in these two studies did engage in perspective-taking, but that they did not use this knowledge during reference production. First of all, as communication theories propose (e.g., Arnold et al. 2013; Clark 1992; Clark and Carlson 1982; Clark and Marshall 1981; Clark and Murphy 1982; Grice 1975), the success of referential communication relies to a great deal on speakers’ ability to regard the perspective of their interlocutor. Without the speakers’ ability to take into account the knowledge and attentional status of the addressee, their reference might not be in line with the required audience design and cooperative principles of communication. Referential communication thus presupposes perspective-taking. Secondly, the data of both speakers’ and addressees’ self-reports support the assumption that speakers were aware of their addressees’ perspective throughout the game. That is, after experiment 1 and 2, both speakers and addressees filled out a perspective-taking questionnaire in which they were explicitly asked to indicate the extent to which speakers had been aware of the addressee’s perspective, and the extent to which they had adjusted their reference production to the addressee’s knowledge and attentional status (see Tables 5 and 7). The results of the self-reports (at least for speakers) correlated with the amount of overspecification. In particular, when speakers reported that they had used redundant information on the task, they had also been more likely to *leak* information about their privileged perspective. Hence, the results of the self-reports and their relation to speakers’ actual reference behavior during the game seem to suggest that speakers were aware which information addressees needed, but that this awareness did not overrule the influence of their egocentric perspective on their subsequent reference production. The self-reports did not reflect speakers’ judgment of their accuracy in taking perspectives, as accuracy should be framed here as the extent to which speakers *leaked* information about their own perspective. By leaking information about their egocentric point of view, speakers provided their addressee with redundant information that could possible cue addressees about speakers’ privileged perspective (Wardlow Lane and Liersch 2012).

One tentative explanation as to why addressees’ perspective was not taken into account when speakers were explicitly made aware of their partner’s informational need is the possibility that speakers relied on other-generated feedback to infer whether their reference was successful or not. If speakers relied on addressees’ contribution to make communication successful (Clark and Brennan 1991; Clark and Krych 2004), they might not have felt the need to correct for perspective mistakes themselves. That is, being under- or overinformative might be less cognitively demanding than integrating the addressee’s perspective before producing a referential utterance. This reliance on addressees’ cueing speakers’ under—or overinformativeness could have allowed speakers’ egocentrism to surface (e.g., Krauss and Fussell 1991; Horton and Keysar 1996). If speakers are able to rely on addressees’ feedback in deciding whether a message was formulated correctly, they do not have to rely on their own cognitive judgement whether their message adheres to addressees’ perspective. In this sense, self-generated feedback—by constantly monitoring whether the to be disclosed information corresponds to addressees’ knowledge and attentional state—is more cognitively taxing than being able to rely on others to detect perspectives mistakes. Speakers are therefore expected to only rely on self-generated cues when other-generated feedback is not available to them (Gann and Barr 2014). This raises the question whether our speakers would have been more attentive to the elicited other-perspective if they were not able to rely on addressees’ collaborative contribution, but were designated to their own judgements. This interesting question could be explored in a research design in which addressees are not able to provide their speaker with feedback.

In the present two studies, speakers leaking information about their own perspective overspecified their referential messages. That is, speakers provided their addressee with information that they did not need to select the intended target. In this sense, addressees did not need to provide their speaker with feedback as speakers’ overspecification did not result in addressees’ misunderstanding. Regardless of the overinformativeness of speakers’ references, addressees were always able to select the intended referent, thereby providing their speaker with positive feedback that the reference had been successful. Perspective mistakes were thus not detected, simply because informational leakage was not regarded as a violation. Though, interesting to note here is that only one addressee did cue his speaker’s overinformativeness during the game. Whereas the remaining addressees remained silent on speakers’ overspecfication during the game, this particular addressee asked his speaker why he included color (/size) information when the target could be distinguished from all the other common-ground figures by just its shape. Prompted by the addressee’s feedback, this speaker reduced his overspecfication after a few more trials, ending up with a reference that adhered to the Gricean maxim of quantity (Grice 1975). Importantly, during debriefing, almost all addressees asked their speaker whether the occluded figure inspired them to include redundant color (/size) adjectives to their referential message. This clearly indicates that overinformativeness communicated relevance (e.g., Davies and Katsos 2013; Engelhardt et al. 2011) to at least these addressees, perhaps allowing these addressees to form an unintended conclusion (e.g., Wardlow Lane and Liersch 2012). This example illustrates the importance of addressees’ collaboration during referential communication. Without cues that speakers are being overinformative, speakers seem to maintain their egocentric reference strategy.

Since the explicit perspective-focus instructions did not reduce speakers’ informational leakage, it could be questioned whether it would not have been better to prime attention to the self- or other-perspective by means of a prompt that was unrelated to the specific stimuli used in the experimental trials (as in Santiesteban et al. 2012; Todd et al. 2011). However, the main objective of the research in this paper was to investigate whether explicit instructions to engage in perspective-taking behavior during the task that presupposes perspective-taking would incite speakers to engage in accurate audience design and, thus, reduce the influence of speakers’ egocentric perspective on reference production. This question is extremely relevant for everyday situations in which people have to engage in (accurate) perspective-taking, without having the opportunity to prime interlocutors with a certain mind-set stimulating perspective-taking prior to the interaction (e.g., Brown 1997, 2010; Fleuridas et al. 1986; Penn 1982; Selvini-Palazzoli et al. 1980; Tomm 1985). The explicit perspective-taking manipulation used in these two experiments should highlight for speakers which information is relevant (Grice 1975) to include in their reference, thereby reducing the extent to which speakers overspecify or, more specifically, leak information about their privileged perspective. Hence, if these explicit instructions to regard the addressee’s mental state that is active during the referential communication do not influence speakers’ audience design or the extent to which they refer to private information, it is unsure whether a prior prompt or primed mind-set that do not address these particular mental states will achieve this.

An interesting topic to discuss is whether the explicit perspective-taking instructions induced a meta-cognitive awareness of perspective overall. That is, speakers being made aware of the figures they themselves were seeing could, as a result, have been made aware of the fact that different perspectives existed, including their awareness of addressees’ divergent perspective. In other words, self-focused speakers could have experienced an altercentric interference—computing the addressee’s perspective alongside the egocentric interpretation—and other-focused could have experienced an egocentric interference—because the egocentric, divergent perspective became more salient (Ferguson et al. 2017; Samson et al. 2010; Samuel et al. 2018). This perspective-switching might weaken any difference we might find in informational leakage between the self- and other-focused speakers, and thus seems plausible considering the insignificant leakage-differences between the two perspective-focus conditions. However, perspective-switching imposes a cognitive burden on interlocutors (Ferguson et al. 2017; Samuel et al. 2018), and this burden would have manifested itself in significant leakage differences between the two perspective-focus conditions (self- and other-focus) and the baseline in which speakers did not receive explicit self- or other-focus instructions. Additionally, since an egocentric interpretation requires less cognitive effort than an allocentric interpretation (Apperly et al. 2009), we are unsure whether self-focused speakers would really have experienced altercentric interferences. Recent findings by Ferguson et al. (2017) validate our initial expectations by showing that interlocutors do not compute the other perspective if they are instructed to remain focused on their egocentric perspective throughout the whole perspective-taking task. Furthermore, the research design of the two experiments presented in this paper did not explicitly invite speakers to switch between perspectives. For instance in contrast to Ferguson et al. (2017) and Samuel et al. (2018), speakers in the self- and other-focus conditions were not imposed with different perspective-switch tasks (i.e., from informed to uninformed addressee), as they received only one perspective-prompt during all 40 trials to either regard the addressee’s perspective (in the other-focus condition) or their own visual perspective (in the self-focus condition). These perspective-focus instructions were also very explicit. That is, speakers explicitly indicated, for each trial, the visual perspective of the addressee, thereby highlighting the common-ground objects including their shared object features addressees were seeing. Although speakers were made aware that addressees were only seeing those three common-ground objects that shared color and size features, speakers were still inclined to refer to the feature of the object that stood out for themselves the most. Even though speakers were aware of the other’s perspective, they did not use this awareness for their subsequent audience design (e.g., Apperly et al. 2010).

An alternative explanation as to why the explicit perspective-taking instructions did not influence speakers’ reference production could be that speakers engaged in a submentalizing process (Heyes 2014) by not representing the visual scene from their addressees’ perspective, but by merely coding which objects were in their addressees’ line of sight. Following this object-centered spatial coding hypothesis (Santiesteban et al. 2014, 2015), speakers could have represented which figures were in front of their addressee, without mentally visualizing these common-ground figures including their shared object-features (i.e., color/size). This would entail that, across the three communicative settings (i.e., baseline, other-focus, self-focus), speakers’ egocentric representation of the figures, especially the contrast between their privileged figure and the target, was the only one that was mentally activated. This could have stimulated speakers to keep referring to the contrasts they themselves were seeing, thereby leaking information about their privileged perspective. However, it is unclear whether speakers’ stimulated visual or an object-centered spatial representation of the common-ground situation would influence speakers’ reference production differently. For both the visual and object-centered spatial representations, speakers’ attention is still focused on the common-ground objects, including the object features that are shared between the objects (size and color). Hence, in both cases, speakers should become aware that referencing the object’s shape (i.e., the only unique property of the object) is enough to allow addressees to successfully identify target. In addition, to our knowledge, the object-centered hypothesis (Santiesteban et al. 2015) has currently only been investigated in the context of language comprehension processing (e.g., Apperly et al. 2010; Keysar et al. 2000, 2003). Future research thus could examine whether similar submentalizing processes during language comprehension are also at work during language production.

The two experiments described in this paper did not replicate the effect of the salience of privileged information on speakers’ information leakage, as evidenced by Wardlow Lane et al. (2006) and Kaland et al. (2011). An important aspect to consider is the experimental setup in which the salience of privileged information was manipulated in Wardlow Lane et al. (2006) and Kaland et al. (2011). To manipulate informational leakage, the researchers in both studies employed a within-subject design in which speakers received different instructions (in separate experimental blocks) as to how to play the referential game. In the privileged block of trials, speakers received the instruction to identify the target so that addressees were able to correctly identify the referents. In the conceal block of trials, speakers received the additional instruction to keep the identity of the hidden figure concealed from addressees. Results of Wardlow Lane et al. (2006) showed that, in the privileged block, the difference between salient (5.4%) and non-salient (0.5%) trials was marginally significant by speakers (*F*_1_, *p* < .06) and significant by items (*F*_2_, *p* < .05). However, when speakers were instructed *not to leak information about the concealed figure* (in the conceal trials), leakage increased to 14.4% on salient trials versus 1.4% on non-salient trials. Kaland et al. (2011) supported these findings and indicated that leakage increased for the salient versus the non-salient trials when speakers received the additional instruction to conceal their private knowledge. The researchers claimed that the additional conceal instruction called for an ironic process (Wegner 1994). When speakers were instructed not to provide information about the hidden figure, they ironically did so because they started to actively think about suppressing leakage behavior, thereby enhancing their attention to their private information. This increased attention to private information helped thoughts of actual leakage to spring to mind. Perhaps this ironic process incited by the conceal instruction caused information to become salient in speakers’ mind, not the other way around. That is, even though the concept of “size” is most salient in speakers’ mind when speakers are presented with conceptually matching objects (e.g., a big privileged circle and a small common-ground circle) versus conceptually mismatching objects (e.g., a big privileged triangle and a small common-ground circle), the conceal instruction could have let speakers to actually pay attention to the salient size contrast presented to them. Are we able to explain why the researchers also found more informational leakage for salient versus non-salient trials on the privileged blocks in which the speakers were only instructed to identify the referents? Perhaps being confronted with different tasks instructions (i.e., identify and conceal) taxed speakers’ working memory, thereby placing a higher task demand on speakers in comparison to a situation in which they are only confronted with one task instruction (i.e., identify) as in our two experiments. It could be that this more cognitively involving experimental setup inspired leakage behavior not only to occur more on salient versus non-salient trials when speakers tried to conceal their private figure, but also on trials in which they only identified the target in common-ground. This would explain why the two experiments in this paper failed to replicate Wardlow Lane et al. (2006) and Kaland et al. (2011) findings. Since our speakers only received one task instruction—to identify the targets—we assume they all paid the same amount of attention to both salient and non-salient trials, probably causing informational leakage to occur for both trials equally. Given our findings, we would suggest that the salience of privileged information is not necessarily derived by the (conceptual) relationship shared between the speakers’ privileged and target figure, but more by the attention this relationship receives. In other words, the size difference for two conceptual matching objects might be salient only when the instruction to conceal this difference increases one’s attention to it.

## Conclusion

This research showed that a stimulated attention to addressees’ perspective did not influence speakers’ audience design during reference production. Even during a relatively easy task and with explicit instructions to take addressees’ knowledge and attentional status into account, speakers were very likely to refer to information that was not known to the addressee.

## Footnotes

1 Speakers’ speech-onset times were also recorded. Since we did not restrict speakers in how they should structure their identifications, large variations in how speakers started their referential utterance existed. This led to the conclusion not to consider this data in our analyses.

2 The proportions of speakers’ informational leakage were not higher in the self-focus condition compared to the baseline, suggesting that explicitly drawing speakers’ attention to their privileged object (i.e., self-focus instructions) did not make them more egocentrically biased than when these instructions were absent (i.e., in the baseline).
